# Improved self-management skills in Chinese diabetes patients through a comprehensive health literacy strategy: study protocol of a cluster randomized controlled trial

**DOI:** 10.1186/1745-6215-15-498

**Published:** 2014-12-20

**Authors:** Wang Hong Xu, Russell L Rothman, Rui Li, Yingyao Chen, Qinghua Xia, Hong Fang, Junling Gao, Yujie Yan, Peng Zhou, Yu Jiang, Yinan Liu, Fangjia Zhou, Wei Wang, Minling Chen, Xiao Yu Liu, Xiao Na Liu

**Affiliations:** Department of Epidemiology, School of Public Health and Key Laboratory of Public Health Safety, Ministry of Education, Fudan University, 138 Yi Xue Yuan Road, Shanghai, 200032 China; Department of Internal Medicine and Pediatrics and Center for Health Services Research, Vanderbilt University Medical Center, Suite 6000, Medical Center East, Nashville, Tennessee 37232-8300 USA; Department of Diabetes Prevention and Control, Shanghai Municipal Center for Disease Control and Prevention, 1380 Zhong Shan Xi Road, Shanghai, 200336 China; Center for Disease Control and Prevention of Changning District, 39 Yun Wu Shan Road, Shanghai, China; Center for Disease Control and Prevention of Minhang District, 965 Zhong Yi Road, Shanghai, 201101 China; Datuan Community Healthcare Center of Pudong New Area, 169 Yong Ding Nan Road, Shanghai, 201311 China

**Keywords:** Cost-effectiveness, Cost-utility, Diabetes self-management, Hemoglobin A1c, Literacy, Numeracy, Self-efficacy, Self-management behaviors

## Abstract

**Background:**

Diabetes self-management often involves the interpretation and application of oral, written, or quantitative information. Numerous diabetes patients in China have limited health literacy, which likely leads to poorer clinical outcomes. This study is designed to examine the efficacy and cost-effectiveness of addressing health literacy to improve self-management skills and glycemic control in Chinese diabetes patients.

**Methods/design:**

This is a cluster randomized controlled trial (RCT) conducted in 20 community healthcare sites in Shanghai, China. Overall, 800 diabetes patients will be randomized into intervention and control arms and will have a baseline hemoglobin A1c (HbA1c) assay and undergo a baseline survey which includes measures of health literacy and diabetes numeracy using revised Chinese versions of the Health Literacy Management Scale and Diabetes Numeracy Test Scale. During the 1-year period of intervention, while the control group will receive usual care, the intervention group will be supplemented with a comprehensive health literacy strategy which includes i) training healthcare providers in effective health communication skills that address issues related to low literacy, and ii) use of an interactive Diabetes Education Toolkit to improve patient understanding and behaviors. Assessments will be conducted at both patient and healthcare provider levels, and will take place upon admission and after 3, 6, 12, and 24 months of intervention. The primary outcome will be the improvement in HbA1c between Intervention group and Control group patients. Secondary outcomes at the patient level will include improvement in i) clinical outcomes (blood pressure, fasting lipids, body mass index, weight, smoking status), ii) patient reported self-management behaviors, and iii) patient-reported self-efficacy. Outcomes at the provider level will include: i) provider satisfaction and ii) intensity and type of care provided. The effects of the intervention will be examined in multivariable general linear models. Both cost-effectiveness and cost-utility analyses will be performed.

**Discussion:**

The main strengths of this study are its large sample size and RCT design, involvement of both patients and healthcare providers, and the long term follow-up (24-months). This project will help to demonstrate the value of addressing health literacy and health communication to improve self-management and clinical outcomes among Chinese diabetes patients.

**Trial registration:**

ISRCTN76130594, Registration date: Sept 22, 2014.

**Electronic supplementary material:**

The online version of this article (doi:10.1186/1745-6215-15-498) contains supplementary material, which is available to authorized users.

## Background

The rising worldwide prevalence of type 2 diabetes mellitus (T2DM) has been very well documented. In China, the prevalence of T2DM tripled between 1980 (about 1.0%) and 1996 (3.2%) [1, 2], and reached 9.7% in 2008 among adults at 20 years old or above [3]. It is estimated that over 92 million adults in China have T2DM; this represents approximately half of the world’s diabetic population and places China at the “*global epicenter of the diabetes epidemic*” [4].

T2DM is a chronic condition that requires patients to follow specific recommendations and prescriptions for the rest of their lives. Due to the lifelong health care it entails and the huge number of diabetic patients, diabetes self-management has become a major component of comprehensive diabetes care around the world. However, less than 50% of recommended guidelines for diabetes management are currently achieved in the United States [5, 6]. In China, where the diabetes self-management strategy has been applied for a couple of years and diabetes education has been suggested to achieve better glycemic control [7, 8], only one-fifth of diabetic patients were reported to achieve hemoglobin A1c (HbA1c) levels of less than 6.5% [9]. One possible reason for this modest effect may be that previous diabetes management did not specifically address issues of health literacy or numeracy. The self-management of diabetes often involves interpretation of quantitative information and the performance of calculations. However, a large number of patients have poor health literacy and numeracy, which lead to incomprehension of basic health information, lower likelihood of receiving preventive care, limited ability to take medications appropriately, higher hospitability, poorer glycemic control status, and worse clinical outcomes.

Literacy has been defined as “*an individual*’*s ability to read*, *write*, *speak*, *and compute and solve problems at levels of proficiency necessary to function on the job and in society*, *to achieve one*’*s goals*, *and develop one*’*s knowledge and potential*” [10]. Patients with low literacy may have trouble in reading prescriptions, following medical instructions, and interacting with the health care system. Patients with low literacy usually have lower disease-specific knowledge, report lower quality of life, and have poorer health related outcomes – even after adjusting for potential confounders such as educational level, insurance, and other factors [10–12]. Numeracy, as an important component of literacy, can be defined as the ability to understand and use numbers in daily life [13]. Numeracy is particularly important to patients with diabetes because diabetes requires self-management skills that rely on mathematics such as counting carbohydrates, interpreting glucose monitoring, applying a sliding scale for insulin, and calculating insulin doses based on carbohydrate intake. These skills require not only basic math skills, but also the ability to apply those math skills in the context of diabetes care (that is, diabetes-related numeracy).

Low literacy is common among patients with diabetes, and appears closely associated with less knowledge of diabetes self-management and worse clinical outcomes [11, 12, 14, 15]. Williams et al. [15] found that 55% of diabetes patients in the United States had inadequate literacy. It was reported that, of patients with inadequate literacy, 50% did not know the symptoms of hypoglycemia, 62% did not know how to treat hypoglycemia, and 42% did not know the normal blood glucose range despite the fact that 73% of the patients had attended previous diabetes education. Among over 400 patients with diabetes, Schillinger et al. [14] observed an independent association of poor literacy with worse glycemic control and higher rates of retinopathy. Most of the studies, to date, on the role of literacy in health care have focused specifically on verbal literacy with little examination of quantitative skills. While there is a strong correlation between verbal literacy and quantitative skills, there are many patients who have adequate verbal literacy but are still unable to use math skills appropriately or are anxious/intimidated about math [16].

Recent studies have demonstrated that providing low literacy materials or low literacy forms of communication can improve patient comprehension for patients with both low and high literacy [17, 18]. A randomized controlled trial (RCT) of a comprehensive disease management intervention demonstrated that literacy was a significant factor in predicting patients’ improvement in HbA1c from the intervention, and suggested that addressing literacy could improve patient outcomes [11]. Two coordinated RCTs performed at two academic medical centers of the United States from 2006 to 2008 rigorously examined the role of addressing both literacy and numeracy, and found that the literacy- and numeracy-sensitive diabetes care can lead to significant improvements in glycemic control, self-efficacy, and other outcomes [19].

So far, very few studies have been specifically designed to address the association of literacy and numeracy with diabetes self-management skills in China. A survey conducted in older diabetes patients in Beijing showed that diabetes knowledge was very limited in this population [20]. In a study conducted in Hong Kong, a negative correlation was observed for health literacy with diabetic control status among diabetes patients [21]. Innovative approaches to diabetes management and education are urgently needed in this population, who has a high prevalence of diabetic complications. Addressing literacy and numeracy through improved healthcare provider communication skills and improved educational materials is a potentially successful strategy. It is an innovative approach to optimize patient understanding, promote shared decision-making, and enhance patient self-efficacy and self-management behaviors. In the current study, we will develop a comprehensive health literacy strategy and evaluate its utility among Chinese diabetes patients. We will report the outcomes of the economic evaluation in a separate article.

The primary outcome of the current study is improvement in HbA1c. We hypothesize that participants randomized to the intervention arm will have a significantly lower HbA1c level when compared to the control group participants. Secondary outcomes are improvements in blood pressure, lipids, self-management behavior, self-efficacy, and other patient outcomes important to improving diabetes care, as well as improved health communication skills and increased satisfaction in health care providers. We hypothesize that patients in the intervention condition will report beneficial changes while the health care providers will be more satisfied with treatment than those in the control condition. A further aim of this study is to evaluate the cost-effectiveness and cost-utility of the intervention. We hypothesize that the intervention will be performed on a large scale in a way that is practical, cost-effective, and sustainable.

## Methods

### Study design

**Figure 1:**
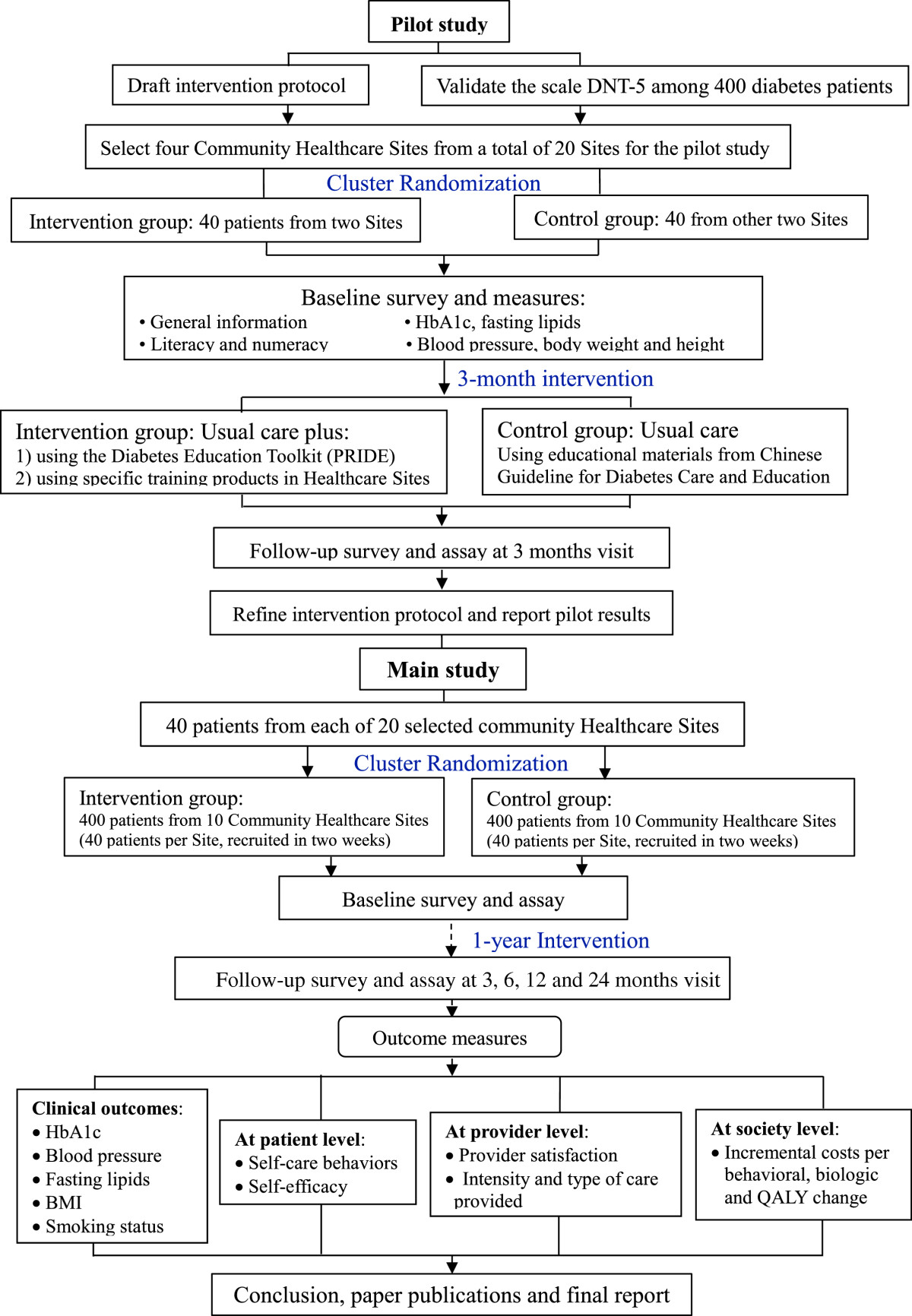
**Flowchart of the study.**

This is a cluster RCT with blinded data analysis (Figure 1). Prior to the trial, we conducted a validation study in 408 diabetes patients in Pudong New area of Shanghai to assess the validity of diabetes-related numeracy test scale in Chinese patients (C-DNT-5). We also performed a pilot study in 80 diabetes patients from eight Community Healthcare Centers in Minhang and Changning district of Shanghai, China, to examine the feasibility and acceptability of intervention and research procedures. We obtained informed consent from each study participant. The results of the two studies have been used to inform the development of the forthcoming trial.

### Setting

A total of 800 diabetes patients will be recruited from eight Community Healthcare Centers in Minhang and Changning district of Shanghai, China. Five clinic sites will be selected from each Center. All clinic sites meet the following criteria: i) at least 20 patients can be recruited per site; ii) at least 2 to 4 physician(s), nurse practitioner(s), or diabetes educator(s) per Center can participate in the intervention; iii) the site agrees to participate for a minimum of 2 years; and iv) the site agrees to be randomized to either arm of the study.

### Inclusion and exclusion criteria for participants

Inclusion criteria at the patient level will include: i) patient has a clinical diagnosis of T2DM; ii) age 18 to 85 years; iii) most recent HbA1c ≥7.5%; iv) patient provides signed informed consent form; and v) patient agrees to participate in the study for the full 2-year duration. Exclusion criteria at the patient level will include: i) poor visual acuity (vision worse than 20/50 using Rosenbaum Pocket Screener); ii) significant dementia or psychosis (by health provider report or chart review); and iii) terminal illness with anticipated life expectancy <2 years.

### Analytical power

According to our previous survey, the mean (SD) of HbA1c among 934 randomly selected diabetes patients was 8.0% (1.4%) [22]. We anticipate a 0.8% improvement in HbA1c level from baseline to 12 months, with a conservative estimate of the SD at 2.0. We performed a simulation study generating follow-up HbA1c using the following formula: Follow-up HbA1c = Baseline HbA1c + β* Group + Site Effect + Provider Effect + δ. Group is a binary variable indicating 0 for control and 1 for intervention group. For 800 patients recruited, we anticipate n = 560 for the final analysis after subtracting 30% loss to follow-up. We will perform multiple imputation for the missing data where all 800 subjects will be used in the analysis; however, our sample size is estimated with the analysis excluding missing observations to provide the most conservative estimate. Assuming 0.8% reduction in HbA1c with the intervention compared with the control group at 12-month follow-up, the proposed analysis will yield analytical power of 100.0% with two-sided 5% significance level.

A simpler sample size calculation than our simulation model would not account for the double clustering design. Using a popularly used formula estimating sample size with single clustering for 40 sites, power is also 100.0% with two-sided 5% significance level to detect the difference of 0.8% in HbA1c.

### Randomization procedure

Randomization will occur at the level of the Community Healthcare Centers. Four Centers (20 clinic sites) will be randomized to receive the intervention and four centers (20 clinic sites) will be randomized to the control condition. To minimize potential imbalance in randomization, we will employ a multivariable score based pair-wise matching method [23] to achieve the optimal balance by location, size, population covered, HbA1c level, and other characteristics of recruited patients including age, gender, insurance status, insulin status, and body weight. We will first weigh covariates according to relevance of potential bias introduced, and then compute a multivariable score using an established algorithm after data are standardized and appropriately weighted. We will then identify four matched pairs, with each pair consisting of two facilities with closest multivariable scores. Randomization will be performed within each pair, with one Center randomized to receive the intervention, and the other to the control condition. This process will be repeated to obtain all possible pair-wise combinations for randomization. We will then assess balance for each covariate as a proportion of imbalance detected over all combinations. When balance is not ideal, the process will be repeated after adjusting the weighting system. After an appropriate weighting scheme is determined, the centers will be paired and a final randomization will be performed. This procedure will ensure more comparable patient populations with respect to clinic characteristics. Randomization at the Center level will help avoid contamination issues at the healthcare provider level.

### Usual care

In the control arm, usual diabetes care will continue according to current national guidelines: conventional clinical consultations, treatment provision according to existing knowledge and at the individual clinician’s discretion, no training in communication skills, and no literacy-/numeracy-sensitive Diabetes Education Toolkit materials.

The control sites will receive a program designed to educate healthcare providers about diabetes care based on the latest diabetes management recommendations and educational materials from the Chinese Guideline for Diabetes Care and Education, which will help to control for Hawthorne effects that might result in improved care.

### Intervention

The intervention will include two main components: i) the Partnership to Improve Diabetes Education Toolkit (PRIDE), a set of plain-language tools to aid provider-patient communication about diabetes management, and ii) a Clear Health Communication Curriculum, a structured training program for healthcare providers to improve diabetes-related counseling communication skills, with specific attention to issues of literacy and numeracy.

#### Diabetes education toolkit (PRIDE)

The PRIDE toolkit will consist of a host of educational materials that have been designed specifically to improve patient understanding and self-management behaviors in patients with lower literacy or numeracy skills. The PRIDE toolkit will be based on the Diabetes Literacy and Numeracy Education Toolkit developed and validated previously [24].

The materials in the PRIDE toolkit are designed specifically to help educate diabetes patients about self-management skills, with 24 educational modules covering all components of diabetes self-management including diet, exercise, foot care, glucose monitoring, and medication management, and enhanced diabetes log sheets that can be shared with patients to help improve their self-management. The modules are written at a low literacy level with many pictures, white space, and other accommodations for low literacy patients. Sample numeracy-oriented accommodations include color coding to help with number hierarchy, the use of insulin pens or syringes with colored marks to aid patients with difficulty measuring insulin, the use of tables and sentences to simplify medication instructions, and simplifying instructions by providing more basic alternatives that do not need as many math skills (e.g., a “plate method” or color coded scoop method instead of carbohydrate counting). For tasks that require math skills, worksheets are provided for patients to practice skills to help gain proficiency.

The toolkit is designed to help accommodate patients of all literacy levels, particularly those with poor literacy or numeracy skills. The PRIDE toolkit also acts as a disease management intervention at the provider and system level by i) acting as a “reminder” system to providers about certain treatment goals, ii) providing a structured approach to addressing self-management goals, including “care algorithms” that promote more systematic approaches to care, and iii) materials addressing important socioeconomic and community level barriers that can impede diabetes care.

Providers in the intervention Centers will be trained in the proper use of the PRIDE toolkit materials and will be expected to use the materials during regular patient-related visits. At each visit, providers will be asked to cover at least two components from the toolkit materials, and to perform and document at least one goal-setting task with the patient. They will share components of the toolkit directly with the patients for the patients to use and take home with them. Providers will be expected to spend approximately 5 to 7 minutes per visit using the toolkit materials with the patients. We anticipate that visits will occur at least every 3 months, in accordance with American Diabetes Association guidelines for diabetes follow-up, or more frequent, as needed. Paper printouts of toolkit materials will be made available in central locations in the intervention Centers for easy access.

#### Clear health communication curriculum for health care providers

Healthcare providers in the intervention arm will be trained in improved health communication skills with a specific emphasis on improving communication to aid patients with poor literacy or numeracy skills. All intervention clinic health care providers (physicians, nurse practitioners, registered nurses, dieticians, and health educators) will gather to obtain an approximately 5 to 6 hour training before the initiation of the intervention. The on-site training of the healthcare providers is essential to promote intervention fidelity and allow rigorous evaluation of the impact of the intervention. To promote provider participation, we will provide certificates with scores of Continuing Medical Education to all providers that participate in the training; we will also provide other nominal incentives for participation (free educational materials, food, pens, etc.).

During the training session, the healthcare providers will receive informational and hands-on training in the following areas: i) diabetes management, ii) introduction to the diabetes education toolkit, iii) clear health communication skills, and iv) application of the diabetes education toolkit using principles of clear health communication.

All intervention health care providers will undergo a post-training certification process to ensure that they have learned the materials. Healthcare providers will meet certification if they accomplish >90% of items listed on a Health Care Provider Certification Checklist at the end of the training day. Providers who fail this certification process will need to participate in additional 1:1 training with the research team until they meet adequate certification criteria. If a provider cannot meet certification requirements, then the participating Center will need to identify additional health care providers to participate in the study.

### Data collection procedures

#### Baseline survey

We will collect baseline characteristics related to the patients and the healthcare providers, which include the following variables:

*Patients*: Age at study entry, gender, date of birth, number of years living in Shanghai, household composition (number of children, marital status, etc.), Mandarin proficiency, health insurance status, employment, income level, years of education, lifestyle (smoking status, dietary habits, and physical activity), literacy level (as measured by the validated Chinese versions of Health Literacy Management Scale) [25], diabetes-related numeracy (as measured by the C-DNT-5), and measures related to clinical history, including years of diabetes, current diabetes medications, history of diabetes education, and glucose monitoring frequency.

*Healthcare Providers*: age, gender, years of education, type of provider (physician, nurse, dietician), Certified Diabetes Educator Status (Yes or No), and satisfaction for health care.

#### Collection of measures

HbA1c and lipids tests will be performed throughout the study using Point-of-Care Equipment available in each Community Health Center (high-performance liquid chromatography for HbA1c, and Automatic Analyzer for Lipids). Self-management activities will be assessed using the previously validated Chinese version of Summary of Diabetes Self-Care Activities [26]. Self-efficacy will be assessed using a scale validated in Chinese adults [26, 27].

The majority of measures will be collected at baseline and 3, 6, 12, and 24 months. The schedule for data collection is summarized in Table 1.

**Table 1:** **Study schedule for data collection**

	Baseline visit	3 month visit	6 month visit	12 month visit	24 month visit
**Patients**					
Characteristics	X				
Health literacy/numeracy	X				
HbA1c tests	X	X	X	X	X
Lipid tests	X			X	X
Systolic and diastolic blood pressure	X	X	X	X	X
Self-management behaviors	X		X	X	X
Self-efficacy	X		X	X	X
**Healthcare Providers**					
Characteristics	X				
Satisfaction with visits	X		X	X	X
Use of intervention (Toolkit) or control (Chinese Guideline for Diabetes Care and Education) materials*	X	X	X	X	X
Certification of training	X	X	X	X	X

*Intervention providers will document after each visit what components of the Diabetes Education Toolkit they used. Control providers will document what Chinese Guideline for Diabetes Care and Education materials they used.

#### Related cost

Fixed and variable costs of the intervention program will be estimated, as well as the short-term and long-term cost-effectiveness of the program. The intervention costs will be subdivided into four categories of costs:

i. *Implementation fixed costs* will include all labor, supply, and space costs of initiating and running the program that are independent of the number of participants in the program. These costs include the cost for program site personnel to attend training sessions before initiation of the program as well as during the program, and the cost of setting up a system to run the program from the clinic site.
ii. *Implementation variable costs* will include all labor, supply, and space costs of running the program that are dependent on the number of program participants. These costs include printing costs for educational materials, labor costs for educating and monitoring the patients, and space costs for storing materials and meeting with patients. All program-related activities that occur within a 2-week time window will be timed using stop watches. These activities will include preparation for the patients’ clinic visits (including gathering educational materials), the clinical encounter, follow-up telephone calls with the patients, and documentation of the visit. These activities will be timed for both the intervention and the control group Centers. The labor cost will be estimated according to the individual’s salary level. Supply and space use for each program activity will also be determined. Costs will be applied to the supplies used based on wholesale acquisition costs. Costs will be applied to clinic visit and storage space used based on standard space overhead rates applied at the program sites. Variation in the fixed and variable implementation costs across the different program sites will be reported.
iii. *Disease*-*related costs* will be derived from health service use data collected during the trial. This will include outpatient drugs prescribed including the dose and duration of therapy, physician visits, laboratory tests, emergency room visits, and hospitalizations. Standard charge will be used to estimate the unit costs for each type of health service, including the medical chart review and extracting, medical insurance system query, and the Physician’s Fee Reference for any healthcare provider costs.
iv. *Patient*-*related costs*, including travel time and costs, total time in the clinic (including time to register, to wait to be seen, to meet with the providers, and to check-out of the clinic), and other incidental costs for each physician and emergency room visit, will be measured using a patient survey administered to all patients visiting the physician or emergency room during the 2-week time window in which the study is being completed. In sensitivity analyses, disability-related costs related to lost work will also be included.

Program development- and research-related costs will not be estimated since they are one-time only costs and would not be incurred when using the program materials in other populations after the end of the study.

### Quality control

A local project office will be set up in each Community Healthcare Center to monitor patient recruitment, consent, and collection of measures. One project officer at each Center will be trained in the recruitment of all patients, including those with lower literacy skills. This process will be rigorously overseen by the Principal Investigator and research team. In addition, the Study Project Coordinator at Fudan University will provide oversight over the project officers. All data will be key-entered in Fudan University.

### Statistical analyses

Comparison of participants by intervention and literacy status will be conducted using χ^2^ tests (for categorical variables) and *t*-tests or Wilcoxon rank-sum tests (for continuous variables). Analysis of covariance will be used to evaluate the improvement in HbA1c levels after adjusting for baseline values. Multivariable general linear models will be used to additionally adjust for other baseline covariates including age, sex, and diagnosis date. Two types of cost-effectiveness ratio (CEA) will be performed: i) a short-term 1-year CEA using a surrogate clinical endpoint, HbA1c, and ii) a long-term lifetime CEA with the outcome measure of quality-adjusted life-years gained. The short-term CEA will use the results of the 1-year cost and clinical analyses to compute the cost-effectiveness ratio, incremental cost per additional person with a reduction of >1% in HbA1c level. The 95% confidence limits for the cost and clinical outcomes measures will be used as input parameter value ranges tested in one-way sensitivity analyses. The long-term CEA will be performed using the Center for Disease Control Diabetes Cost-Effectiveness Group Model that evaluates the cost-effectiveness of intensive glycemic control, blood pressure control, and cholesterol control in people with diabetes [28]. All analyses will be performed using SAS version 9.13.

Approval was obtained from the Medical Ethics Committee of School of Public Health, Fudan University (IRB00002408 & FWA00002399) (registration number: 2013-06-0451). The involved local medical ethics committees in the Center for Disease Control and Prevention of Changning District and the Center for Disease Control and Prevention of Minhang District agreed with this approval.

## Discussion

This article describes the study protocol of a cluster RCT examining the efficacy and cost-effectiveness of addressing health literacy and numeracy to improve self-management skills and glycemic control in Chinese diabetes patients. The results of the RCT will rigorously demonstrate that addressing poor health literacy and numeracy in practical, “real world” settings can significantly improve self-management skills and clinical outcomes for patients with T2DM. The research will also demonstrate that this type of intervention can be performed on a large scale in a way that is practical, cost-effective, and sustainable.

We hypothesize that participants randomized to the intervention arm will have significant improvements in HbA1c, blood pressure, lipids, self-management behavior, self-efficacy, and other patient outcomes important to improving diabetes care when compared to the control group participants. We also hypothesize that i) patient health literacy or numeracy will be a significant effect modifier on the study impact –among patients with lower literacy or numeracy skills, those receiving the intervention will have more significant improvement in HbA1c level and other outcomes; ii) health care providers in the intervention group will report improved health communication skills and increased knowledge, and satisfaction compared to providers in the control group; iii) the intervention will be of modest program costs and will be cost-effective in the long-term by leading to improved patient clinical outcomes, decreased complications, and decreased use of high-cost services such as emergency room visits and hospitalizations.

To date, very few studies have been specifically designed to address the association of literacy and numeracy with diabetes self-management skills in Chinese diabetes patients. If the study hypotheses are confirmed, the toolkit and the healthcare provider training curriculum and material could be more widely distributed and applied in diabetes health care, reducing incidence of diabetic complications and premature mortality, and limiting the costs due to diabetic complications.

The strengths of this study include data are collection in a naturalistic setting (the age of patients range from 18 to over 80 years, involvement of both patients and healthcare providers) and the examination of hypotheses in a RCT design. A further strength is the size of the study, in which healthcare providers and diabetes patients from urban and rural communities are included. Moreover, 24-month follow-up of participants will provide an opportunity to evaluate the long-term effect of the intervention.

A possible limitation of the study is the short 1-year intervention, which may not be long enough to observe the effect of the intervention on occurrence of diabetes complications. In this study, the main outcome is the reduction in HbA1c level, which is a surrogate marker for long-term outcomes. Studies such as the DCCT and the UKPDS demonstrate that reducing HbA1c by ~0.7% can reduce micro-vascular complications by as much as 40 to 60%. Lowering HbA1c in T2DM also can decrease the absolute risk of developing coronary heart disease by 5 to 17% and all-cause mortality by 6 to 15% [29]. We can assume that if 1-year intervention can decrease HbA1c level significantly, it will improve long-term outcomes of diabetes patients. We also include blood pressure and lipid control as clinical outcomes. Second, the 1-year intervention may lead to a high drop-out rate. To prevent drop-out, local research assistants/nurses will help patients to complete the questionnaires and will make appointments for follow-up assessment. Long-term incentives will also be applied for both the healthcare providers and the patients. Third, although all Community Healthcare Centers in Shanghai provide diabetes care services according to the Guideline of Chinese Diabetes Prevention and Control, the usual care in the Centers of Minhang district is somewhat different from that in Changning district. In order to overcome this limitation, we will ensure the matched pair of Centers from a same district. Moreover, the patients will be from the Minhang district and the Changning district, two of a total of 13 districts in Shanghai, China. This may arouse concerns on the representation of the sample patients and extrapolation of our results to other patients. From a generalizability perspective, this study will recruit a diverse population across urban and rural Shanghai, and may be generalizable to other areas in China. Finally, given that Shanghai is one of the most economically developed large cities in China, whose residents have a relatively higher educational level, the results derived from our population may underestimate the effect of addressing health literacy and numeracy to improve self-management skills and glycemic control in Chinese diabetes patients. Nevertheless, it is plausible that the interventions, if effective in our population, would work in other populations.

## Trial status

Patient recruitment is ongoing.

## Authors’ original submitted files for images

Below are the links to the authors’ original submitted files for images. Authors’ original file for figure 1

## Abbreviations

CEA: Cost-effectiveness ratio
C-DNT-5: Chinese version of Diabetes Numeracy Test Scale
HbA1c: Hemoglobin A1c
PRIDE: Partnership to Improve Diabetes Education Toolkit
RCT: Randomized controlled trial
T2DM: Type 2 diabetes mellitus.
