# Supplementary figures and images for: Improved self-management skills in Chinese diabetes patients through a comprehensive health literacy strategy: study protocol of a cluster randomized controlled trial
Source: Trials. 2014 Dec 20;15:498. doi: 10.1186/1745-6215-15-498 (PMC4307742; doi:10.1186/1745-6215-15-498)

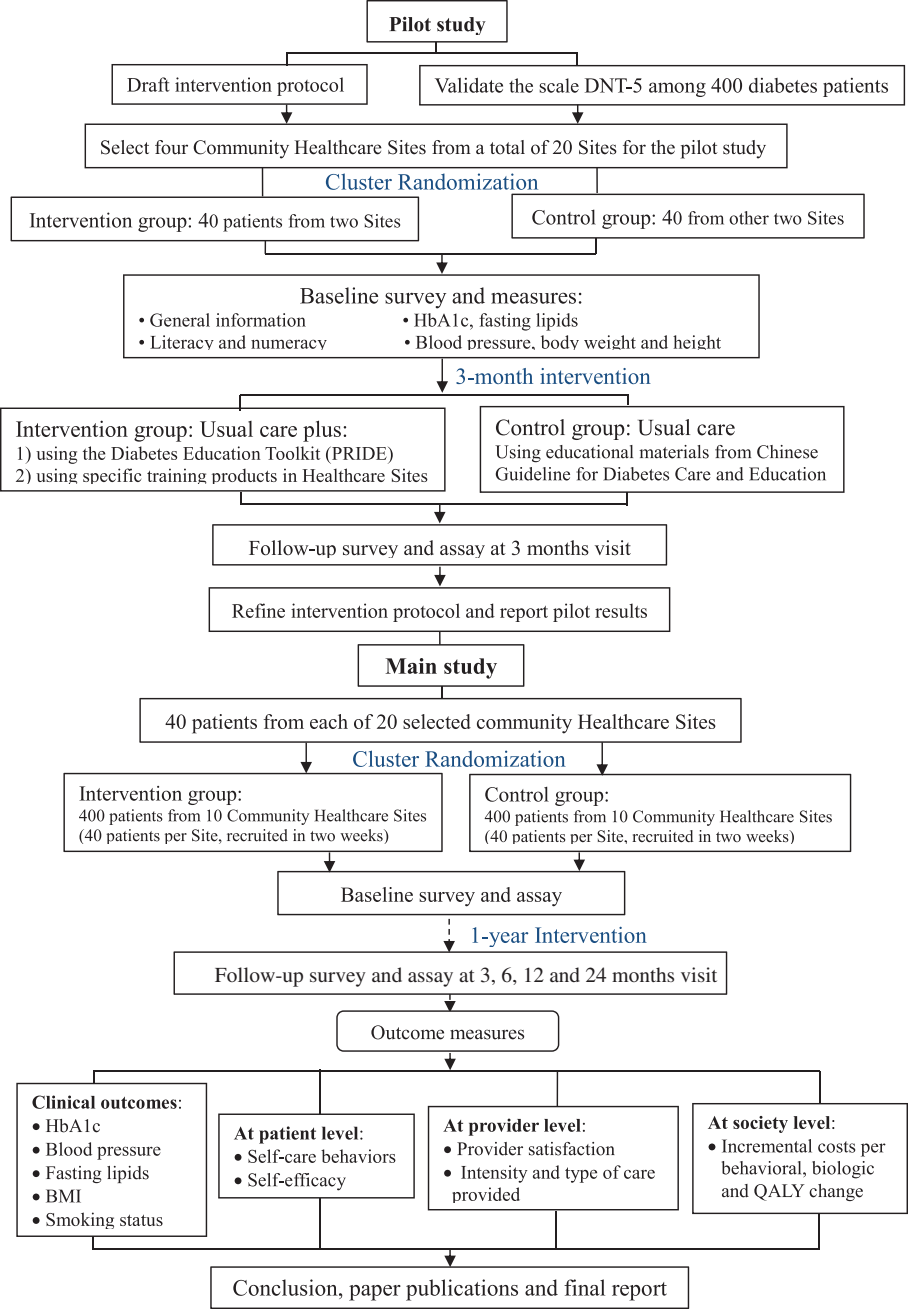

Supplement: Supplementary file 1 — Authors’ original file for figure 1 [file 13063_2014_2370_MOESM1_ESM.pdf]
